# Gaining consensus on expert rule statements for acute respiratory failure digital twin patient model in intensive care unit using a Delphi method

**DOI:** 10.17305/bb.2023.9344

**Published:** 2023-12-01

**Authors:** Amy J Montgomery, John Litell, Johnny Dang, Laure Flurin, Ognjen Gajic, Amos Lal, on behalf of Digital Twin Platform for education, research, and healthcare delivery investigator group

**Affiliations:** 1 Department of Internal Medicine, Mayo Clinic, Rochester, USA; 2 Department of Emergency Critical Care, Abbott Northwestern, Minneapolis, USA; 3 Department of Neurology, Cleveland Clinic, Cleveland, USA; 4 Department of Laboratory Medicine and Pathology, Mayo Clinic, Rochester, USA; 5 Pulmonary and Critical Care Medicine, Mayo Clinic, Rochester, USA

**Keywords:** Digital twin, intensive care unit (ICU), respiratory failure, hypoxia, consensus, critical care, Delphi

## Abstract

Digital twin technology is a virtual depiction of a physical product and has been utilized in many fields. Digital twin patient model in healthcare is a virtual patient that provides opportunities to test the outcomes of various interventions virtually without subjecting an actual patient to possible harm. This can serve as a decision aid in the complex environment of the intensive care unit (ICU). Our objective is to develop consensus among a multidisciplinary expert panel on statements regarding respiratory pathophysiology contributing to respiratory failure in the medical ICU. We convened a panel of 34 international critical care experts. Our group modeled elements of respiratory failure pathophysiology using directed acyclic graphs (DAGs) and derived expert statements describing associated ICU clinical practices. The experts participated in three rounds of modified Delphi to gauge agreement on 78 final questions (13 statements with 6 substatements for each) using a Likert scale. A modified Delphi process achieved agreement for 62 of the final expert rule statements. Statements with the highest degree of agreement included the physiology, and management of airway obstruction decreasing alveolar ventilation and ventilation-perfusion matching. The lowest agreement statements involved the relationship between shock and hypoxemic respiratory failure due to heightened oxygen consumption and dead space. Our study proves the utility of a modified Delphi method to generate consensus to create expert rule statements for further development of a digital twin-patient model with acute respiratory failure. A substantial majority of expert rule statements used in the digital twin design align with expert knowledge of respiratory failure in critically ill patients.

## Introduction

Digital twin technology is an emerging concept that has shown tremendous potential in transforming healthcare delivery and medical research. Essentially, a digital twin is a virtual depiction of a physical entity, system, or process that can be used to mimic, analyze, and optimize its behavior in real time [1]. In healthcare, digital twin technology can be applied to model and simulate different aspects of the human body, such as organs, tissues, and even entire biological systems, providing healthcare providers with valuable insights into disease mechanisms, treatment efficacy, and personalized patient care. Recent developments in computational power, machine learning, and big-data analytics have made it possible to create more sophisticated and accurate models of the human body, thus advancing the emerging domain of the development and refinement of digital twins. Although still in its infancy, the technology has the potential to revolutionize medical research by providing a powerful tool for predicting the behavior of biological systems, simulating disease progression, and designing more effective treatment options.

Moreover, digital twin technology can also be used to enhance clinical decision-making by providing end users (learners, trainees, and bedside clinicians) with real-time insights into patient conditions, enabling them to make more accurate diagnoses and design personalized treatment plans. As technology continues to evolve and become more sophisticated, it is estimated to play a progressively more important role in transforming the healthcare industry. Artificial intelligence (AI) is additionally on the rise within the field of healthcare. For example, the Archimedes model has been previously designed based on the physiology and interventions used to manage diabetes mellitus (DM) [2]. The DM model simulates multiple organ systems, each with specific functions that can be affected by various disease states. It is intended to be used as a patient simulation to provide “clinical” experience, reach answers sooner and less expensively than empirical studies, or for situations that would put the live patient at risk for harm. The DM model has been successfully validated in clinical simulation trials.

In contrast to creating a model to predict outcomes of a chronic disease process that takes many years to develop complications, a critical care digital twin will be refined and validated in a data-rich environment with a rapid turnaround time for the interventions and associated effects. The physiology simulator, HumMod, has been used in virtual patients’ medical education for chronic illness management [3]. These simulation models can introduce a new facet to medical education, the science of simulation, and clinical practice by improving knowledge, competency, and skill level; and ultimately minimizing clinical errors [3]. As a clinical practice tool, a patient digital twin will make available the bedside providers to preview how the different organ systems interact in causing a clinical effect. This will provide the prospect to test the consequences of various interventions in silico without subjecting an actual patient to potential harm.

The intensive care unit (ICU) digital twin, a form of AI, is a virtual equivalent to critically ill patients that mirrors the interactions and effects of the intervention of the major organ systems [4]. The directed acyclic graph (DAG) approach represents these relationships between patient factors and treatments. A causal DAG is a visual illustration of interacting concepts and variables represented by multiple nodes and edges. The variables are connected with arrows showing the hypothesized causal effect’s direction [5]. DAGs serve as the foundation for AI models as diagrams that represent pathophysiology concepts based on knowledge from content experts [6]. Unidirectional arrows are used to simplify complex causal effects by representing individual components [7].

Our group has successfully worked on iteratively developing, prospective verifying, and testing the preliminary performance of a DAG-based causal AI model to predict the treatment response during the first 24 hours of sepsis [8]. While moving away from the associative AI (black box) models, our previous work has emphasized the causal pathways to design a model which end-users would trust. The use of black-box or associative AI has been attempted with limited real-world success, and some of the examples, such as IBM’s Watson, have demonstrated its limitations when it comes to clinical decision-making in real-world patients. Although the model performed well in the in-silico environment, the performance lacked the fidelity promised in preliminary studies [9].

The interactions represented by DAGs were used to compile the “expert rule” book. The expert rules are based on current best practices based on content experts’ clinical experiences and respiratory failure’s fundamental pathophysiology [10]. Expert rules define the effects that variables have on each other, and various causes (interventions and interactions) lead to specific effects on organ systems reflected by clinical markers (i.e., increased heart rate, decreased urine output, decreased Glasgow Coma Scale, etc.).

Graphical representation of these concepts has been captured in the development of several DAGs for a multi-organ system. The currently presented oxygenation–ventilation DAG is one such example. Based on the expert rule book, program coding has been done to demonstrate the response of interventions in a virtual patient [7]. Building on the previous work, this project focuses on oxygenation–ventilation rules in acute respiratory failure in critically ill patients in the medical ICU. This project demonstrates the use of a modified Delphi process for establishing agreement on the interactions that ICU interventions have with outcomes of respiratory physiology. Preliminary data from this project were presented at the American College of Chest Physicians Annual Congress in 2022 [11] and the Society of Critical Care Medicine Annual Congress in 2023 [12].

## Materials and methods

**Figure 1.**
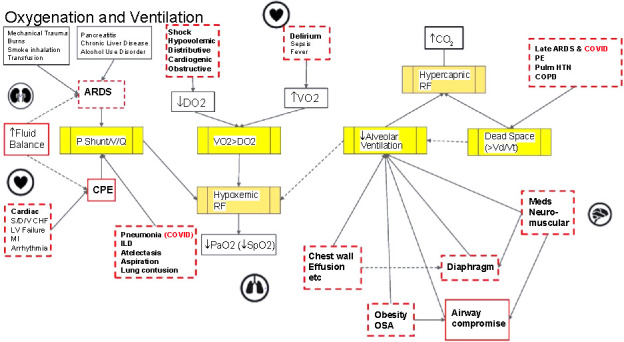
**Directed acyclic graph for oxygenation and ventilation of critically ill patients.** Solid arrows represent cause leading to effect in the direction of the arrow. Red boxes represent concepts. Solid red boxes represent actionable factors, while dashed red boxes represent semi-actionable factors. ARDS: Acute respiratory distress syndrome; V/Q: Ventilation/perfusion; CPE: Cardiogenic pulmonary edema; S/D/V CHF: Systolic/diastolic/biventricular congestive heart failure; LV: Left ventricular; MI: Myocardial infarction; ILD: Interstitial lung disease; DO2: Arterial oxygen delivery; VO2: Oxygen uptake; RF: Respiratory failure; PaO2: Arterial partial pressure of oxygen; CO_2_: Serum carbon dioxide; PE: Pulmonary embolism; Pulm HTN: Pulmonary hypertension; COPD: Chronic obstructive pulmonary disease; Vd/Vt: Physiologic dead space/tidal volume; OSA: Obstructive sleep apnea.

A steering committee of clinicians within internal medicine, critical care, emergency medicine, and pulmonary critical care medicine from two institutions drafted elements of acute respiratory failure pathophysiology using a DAG and resultant expert statements describing accompanying ICU clinical processes (Figure 1). Additional physicians iteratively refined these statements within one of the institutions. Once editing was completed and reviewed by the initial research group, the statements were used to create the first round of the Delphi survey.

Email invitations to participate in the Delphi process were sent to an international group of 34 experts in critical care and pulmonary physiology. Experts were invited to voluntarily participate in this multinational Delphi process with an intent of satisfying the requisites of diversity in sex (males and females), years of experience (ranging 5 years to 30 years), specialties (pulmonary-critical care, emergency-critical care, critical care medicine, etc.), and geographical distribution [13]. Upon acceptance of the invitation, a modified Delphi panel of 30 international experts was instituted. Surveys were prepared using REDCap and administered to participants via email using the REDCap survey link [14].

### Ethical statement

The study was approved by Mayo Clinic IRB (IRB # 18-000831). Informed consent was obtained from all survey participants to participate and share their opinions. All methods were carried out in accordance with relevant guidelines and regulations.

### Statistical analysis

The initial Delphi survey comprised 13 main statements, each statement had six substatements, for a starting total of 78 survey questions. Panelists participated in three Delphi rounds to assess the agreement using a 6-point Likert scale (with zero being “completely disagree” and six being “completely agree”). The consensus was defined as ≥80% agreement (selection of 5 [“agree”] or 6 [“strongly agree”]). Three rounds of Delphi were completed to determine expert consensus among the statements. Statements reaching an agreement during the first round were excluded from subsequent rounds. Statements with less than 80% selection of a five or a six were reviewed by the steering committee and were either modified, unchanged, or excluded from the following rounds of Delphi. The process was repeated to prepare for Delphi’s third and final round.

## Results

A convenience sample of 34 experts was sent an invitation to participate in this multinational study through the Discovery Research Network, Society of Critical Care Medicine. Thirty (88%) from 5 countries and 6 states within the U.S. participated in the Delphi process (Table 1), and 22 (73%) completed the entire Delphi process (all 3 cycles). The median age was 42 years (interquartile range 39–48 years) for the final panel of experts. The number of female experts in the final panel was 7 (31.8%) (Table 1). The final panel included 18 (81.8%) experts in critical care, 7 (31.8%) in internal medicine, 8 (36.4%) in pulmonary medicine, 2 (9.1%) in anesthesiology, 7 (31.8%) in emergency medicine, 2 (9.1%) in infectious diseases, 1 (4.5%) in cardiology, 2 (9.1%) in pediatrics, and 2 (9.1%) in pharmacy.

**Table 1:** Demographics of the expert Delphi panel

**Variables**	**Total, *N* ═ 22**
*Sex*	
Male	15 (68.2%)
Female	7 (31.8%)
*Years in practice*	
5–10	2 (9.1%)
11–15	9 (40.9%)
16–20	7 (31.8%)
21–25	3 (13.6%)
26–30	1 (4.6)
*Geographic distribution*	
United States of America	7 (31.8%)
Japan	12 (54.4%)
Peru	1 (4.6%)
Serbia	1 (4.6%)
Saudi Arabia	1 (4.6%)

**Figure 2.**
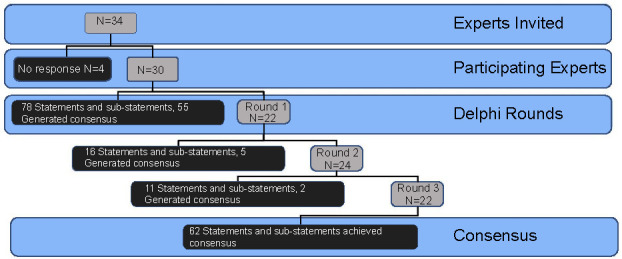
**Flow diagram of Delphi process steps.** N: Number of experts.

Three rounds of surveys were completed between February 9 and March 30, 2022. The first Delphi round included statements related to the pulmonary physiology of acute respiratory failure affecting critically ill patients, e.g., pulmonary edema, shock, acute respiratory distress syndrome (ARDS), airway obstruction, restrictive lung disease, and ventilation–perfusion mismatch (V/Q mismatch). The first round included 13 main statements with 78 total questions (each statement included 6 questions regarding direction, timing, intensity, probability, contingencies, and therapeutic implication). Discussion of results from the first round led to the exclusion of 7 questions, and remaining 71 questions (statements and substatements) were analyzed for the remainder of the modified Delphi process (Table 2). Thirty experts participated in the first round, 8 experts provided partial completion with 22 completed responses. These experts were all invited to participate in the second round (Figure 2).

**Table 2:** Final table of Delphi statements accompanied by which round of Delphi reached consensus

**Statement**	**Rounds Until Consensus**
**Question 1**	
Positive net fluid balance worsens both cardiogenic pulmonary edema and ARDS (non-cardiogenic/injury pulmonary edema) by contributing to extravascular lung water, increased pulmonary shunt, and ventilation/perfusion mismatch.	
*Direction:* In patients with pulmonary edema/ARDS, increased positive fluid balance leads to decreased oxygenation.	1
*Intensity:* The effect is higher with a higher net positive fluid balance.	1
*Timing:* Delayed effect after 12 hours.	3
*Probability:* High.	1
*Contingencies:* Decreased ability to adapt to increased fluid balance (such as heart/renal/liver failure or shock) will potentiate the effect of increased fluid balance.	1
*Therapeutic implication:* Diuresis, renal replacement therapy (CRRT, HD), noninvasive ventilation, intubation, and mechanical ventilation.	1
**Question 2**	
Parenchymal lung diseases will create pulmonary shunt and V/Q mismatch which can cause hypoxemic respiratory failure.	
*Direction:* Increased pulmonary shunt and/or V/Q mismatch will lead to hypoxemia.	1
*Intensity:* The effect increases with worsened shunt or mismatch.	1
*Timing:* Delayed.	2
*Probability:* High.	1
*Contingencies:* Coexisting lung disease (e.g., COPD), pulmonary embolism, mucus plugging, and congenital heart diseases can potentiate the effects of shunt and/or mismatch.	1
*Therapeutic implication:* Treat the underlying cause (anticoagulation for pulmonary embolism, bronchoscopy with mucus removal for mucus plug).	1
**Question 3**	
*Original statement:* Shock and increased oxygen consumption (VO2) with unchanged oxygen delivery (DO2) cause hypoxemic respiratory failure.	
*Final statement:* Shock and/or O_2_ mismatch contributes to or worsens hypoxemic respiratory failure.	
*Direction:* Increased VO2 causes decreased venous oxygen saturation (SvO2) and worsens hypoxemia.	No consensus
*Intensity:* Higher oxygen demand situations, such as severe shock, seizure, and hypermetabolic states (fever) will worsen hypoxemia.	2
*Timing:* 0–3 hours.	No consensus
*Probability:* High.	No consensus
*Contingencies:* Conditions that impair oxygenation (underlying lung disease, pulmonary shunt, V/Q mismatch, ARDS, or cardiogenic pulmonary edema) will potentiate the effect.	1
*Therapeutic implication:* Treat the underlying cause (antibiotics for bacterial pneumonia, steroid use for COVID-19, antiviral agents for influenza pneumonia, fluids for sepsis), respiratory support and intubation, anticonvulsants, neuromuscular blockade, infection source control.	1
**Question 4**	
*Original statement:* ARDS can increase dead space.	
*Final statement:* ARDS can increase dead space by vascular injury and/or overdistension (high PEEP).	
*Direction:* Increased extent or severity of injury increases VO2 and dead space.	1
*Intensity:* Higher effect with worse injury.	1
*Timing:* 12–24 hours.	No consensus
*Probability:* High.	1
*Contingencies:* Conditions that impair oxygenation (underlying lung disease, pulmonary shunt, V/Q mismatch, or cardiogenic pulmonary edema) and high PEEP will potentiate the effect.	No consensus
*Therapeutic implication:* Treat the underlying cause of lung injury, supplemental oxygen (nasal cannula, face mask, high flow nasal oxygen), or mechanical ventilation.	3
**Question 5**	
Upper airway obstruction (e.g., loss of tone due to sedation) and/or lower airway obstruction (e.g., acute COPD exacerbation) cause decreased alveolar ventilation.	
*Direction:* Increased airway compromise causes decreased alveolar ventilation.	1
*Intensity:* Higher effect with increased amount of airway compromise.	1
*Timing:* Variable (could be immediate or delayed).	1
*Probability:* High.	1
*Contingencies:* Additional factors that reduce alveolar ventilation (chest wall or diaphragm abnormalities, positive fluid balance, pre-existing lung disease, etc.) will potentiate the effect. Patients with COPD who get timely bronchodilators and steroids can have improved ventilation.	1
*Therapeutic implication:* Management of underlying cause (naloxone for opioid overdose), pulmonary hygiene for secretion burden bronchodilators, inhaled or systemics steroids, respiratory support, and intubation.	1
**Question 6**	
Diaphragmatic or chest wall abnormalities (neuromuscular disorders, pleural effusion) can lead to reduced alveolar ventilation.	
*Direction:* Decreased diaphragm functioning or diaphragmatic weakness, decreased chest wall expansion (obesity, rib fractures, accessory respiratory muscle fatigue) will decrease alveolar ventilation.	1
*Intensity:* Higher effect with higher diaphragmatic dysfunction, low intensity for chest wall abnormalities.	1
*Timing:* Immediate.	No consensus
*Probability:* High if diaphragm dysfunctions, low if the sole underlying issue is chest wall abnormalities.	2
*Contingencies:* Additional factors that reduce alveolar ventilation (positive fluid balance, heart failure) will potentiate the effect.	1
*Therapeutic implication:* Management of underlying cause (IVIG or plasmapheresis for myasthenia gravis or Guillain Barre syndrome, pain management in chest wall injury, thoracentesis/diuresis for pleural effusions, naloxone for opioid overdose), positive pressure ventilation, supplemental oxygen (nasal cannula, face mask, high flow nasal oxygen), or mechanical ventilation.	1
**Question 7**	
Decreased ventilation or increased dead space volume can lead to hypercapnic respiratory failure.	
*Direction:* Decreased ventilation or increased dead space will increase blood carbon dioxide levels.	1
*Intensity:* Higher effect with higher area affected (decreased ventilation or decreased gas exchange).	1
*Timing:* Variable (could be immediate or delayed).	1
*Probability:* High.	1
*Contingencies:* Hypercapnia can be acute (central nervous system depression, mechanical defects, respiratory fatigue, acute worsening of bronchoconstriction in COPD exacerbation) or chronic (stable COPD, obesity hypoventilation syndrome, causes of intermittent airway obstruction).	1
*Therapeutic implication:* Treat the underlying cause and provide noninvasive or invasive mechanical ventilation.	1
**Question 8**	
Opioids and other respiratory depressants/sedatives can suppress respiratory drive and cause decreased alveolar ventilation, which can lead to hypercapnic respiratory failure and acidosis.	
*Direction:* Opioids and respiratory depressant medications suppress respiratory center leading to decreased respiratory rate, decreased alveolar ventilation and hypercapnic acidosis (high CO_2_, low pH).	1
*Intensity:* The effect is higher with higher doses.	No consensus
*Timing:* The effect is immediate for IV and delayed for oral administration.	1
*Probability:* High.	1
*Contingencies:* Conditions that decrease tidal volume (such as obesity, neuromuscular disease, kyphosis) will potentiate the effect; higher doses can cause airway compromise.	1
*Therapeutic implication:* Antidote (naloxone), respiratory support (noninvasive ventilation), and intubation (if airway compromise).	1
**Question 9**	
Traumatic injuries can cause ARDS.	
*Direction:* Increased traumatic forces increase risk of ARDS.	1
*Intensity:* High.	2
*Timing:* Delayed by 12 to 24 hours.	2
*Probability:* Moderate.	No consensus
*Contingencies:* Additional causes of lung injury (infection), multiple rib fractures or cardiopulmonary edema will potentiate the effect.	1
*Therapeutic implication:* Lung protective ventilation, respiratory support (nasal cannula, face mask, high flow nasal oxygen), mechanical ventilation, prone ventilation, ECMO.	1
**Question 10**	
Acute heart failure, myocardial infarct, or arrhythmia can lead to elevated filling pressures and cardiogenic pulmonary edema.	
*Direction:* Increased severity of cardiac abnormality (decreased ejection fraction) will increase cardiopulmonary edema.	1
*Intensity:* High.	1
*Timing:* Variable, heart failure will be delayed to point of decompensating, whereas infarct or arrhythmia may cause an immediate effect.	1
*Probability:* Moderate.	1
*Contingencies:* Additional causes of pulmonary edema (lung infection or injury) will potentiate the effect, concomitant renal failure will increase the chances of developing cardiogenic pulmonary edema.	1
*Therapeutic implication:* Management of underlying cause (cardiac stent placement for acute infarct, antiarrhythmics or cardioversion for arrhythmias), diuresis, positive pressure ventilation (non-invasive or invasive).	1
**Question 11**	
Parenchymal pulmonary infiltrates (pneumonia, atelectasis, cardiogenic and non-cardiogenic-ARDS pulmonary edema) can cause V/Q mismatch.	
*Direction:* Parenchymal pulmonary infiltrates will increase the amount of pulmonary shunt or mismatch.	1
*Intensity:* High.	1
*Timing:* Immediate.	1
*Probability:* High.	1
*Contingencies:* Additional causes of pulmonary shunting/V/Q mismatch (pneumonia, atelectasis, ARDS, pulmonary embolism, acute decompensated heart failure, AKI with fluid overload) will potentiate the effects.	1
*Therapeutic implication:* Diuresis, renal replacement therapy (CRRT, HD) respiratory support (supplemental oxygen, nasal cannula, face mask, high flow nasal oxygen), and intubation if mismatch (not complete shunt).	1
**Question 12**	
Acute blood loss can lead to decreased oxygen delivery and shock even without an increase in oxygen consumption.	
*Direction:* Decreased oxygen delivery will lead to a compensatory increase in oxygen extraction.	Excluded
*Intensity:* Higher effect with higher blood loss.	Excluded
*Timing:* Delayed.	Excluded
*Probability:* Moderate.	Excluded
*Contingencies:* In the setting of increased shunt (pneumonia, edema, atelectasis, ARDS), increase in VO2 will lead to worsening arterial hypoxemia.	Excluded
*Therapeutic implication:* Treat the underlying cause, blood transfusion, supplemental oxygen (nasal cannula, face mask, high flow nasal oxygen), and mechanical ventilation.	Excluded
**Question 13**	
Obesity hypoventilation and OSA involve reduced chest wall compliance and upper airway obstruction, which can decrease alveolar ventilation.	
*Direction:* Increased body weight increases airway compromise, decreased chest wall and overall respiratory system compliance and decreases alveolar ventilation.	1
*Intensity:* Low.	Excluded
*Timing:* Delayed.	No consensus
*Probability:* High.	1
*Contingencies:* Additional causes of airway compromise (COPD, OSA, medications) will potentiate the effect.	1
*Therapeutic implication:* Positive pressure ventilation, appropriate positioning, respiratory support.	1

ARDS: Acute respiratory distress syndrome; V/Q: Ventilation–perfusion; PEEP: Positive end-expiratory pressure; CRRT: Continuous renal replacement therapy; HD: Hemodialysis; COPD: Chronic obstructive pulmonary disease; IVIG: Intravenous immunoglobulin; IV: Intravenous; ECMO: Extracorporeal membrane oxygenation; AKI: Acute kidney injury; OSA: Obstructive sleep apnea.

The agreement was achieved on 60 (84.5%, 60 out of 71 final statements and substatements) expert statements after completing 2 rounds. After completing the third round, the agreement increased to 62 (87%, 62 out of 71 final statements and substatements). Statements that reached the highest agreement included the underlying pathophysiology and clinical management of airway obstruction reducing alveolar ventilation and the effects of alveolar infiltrate on V/Q matching. The lowest agreement was found for the statements relating the association between shock and hypoxemic respiratory failure due to heightened oxygen consumption and ARDS worsening dead space.

In the review of the first-round results, the statement “acute blood loss can lead to decreased oxygen delivery and shock even without an increase in oxygen consumption” was removed due to concept redundancy. Four statements from the initial survey reached an agreement on all six associated questions. These statements were: upper airway obstruction (e.g., loss of tone due to sedation) and/or lower airway obstruction (e.g., acute chronic obstructive pulmonary disease [COPD] exacerbation) cause decreased alveolar ventilation; decreased ventilation or increased dead space volume can lead to hypercapnic respiratory failure; acute heart failure, myocardial infarct, or arrhythmia can lead to elevated filling pressures and cardiogenic pulmonary edema; and pulmonary parenchymal infiltrates (pneumonia, atelectasis, and cardiogenic and non-cardiogenic-ARDS pulmonary edema) can cause V/Q mismatch.

## Discussion

Delphi research methodology is a structured, iterative process that utilizes expert opinions to make forecasts or judgments about a particular topic which needs input from the experts in the field to provide more certainty in the collective intelligence. A Delphi research project can be conducted on a multinational level as we have described in the methodology section, which involves soliciting expert opinions from multiple countries or regions. This project reports the utility of a modified Delphi process to reach expert consensus for statements regarding respiratory pathophysiology in the medical ICU that will serve as the guidelines for the ICU digital twin AI model. Similar Delphi survey has been completed and published by the authors for confirming the expert rules in the neurocritical care setting for the management and the pathophysiology of acute stroke care [15]. This patient level digital twin model subsequently will be validated compared to real-time ICU patient data using a methodology previously published by our group [8]. The first Delphi round included statements of pulmonary physiology affecting critically ill patients, e.g., pulmonary edema, hypoxemic and hypercapnic respiratory failure, shock, ARDS, airway obstruction, restrictive lung disease, and V/Q mismatch. After the completion of two rounds, the agreement was 60 (84.5%), which increased to 62 (87%) of the expert statements at the end of 3 rounds.

The majority of questions that did not reach agreement after three rounds were associated with the statements “shock and increased oxygen consumption (VO2) with unchanged oxygen delivery (DO2) cause hypoxemic respiratory failure” and “ARDS can increase dead space.” During review and edits in the modified Delphi process, these statements were changed to “shock and/or O_2_ mismatch contributes to or worsens hypoxemic respiratory failure” and “ARDS can increase dead space by vascular injury and/or overdistension (high positive end-expiratory pressure PEEP).” Oxygen utilization during various shock states is controversial in critical care and of unclear etiology [16]. According to our expert panel, oxygen consumption and delivery mismatch do not necessarily correlate with hypoxemic respiratory failure. However, some research has expressed the opinion that impaired oxygen delivery, such as in patients with systemic inflammation and organ dysfunction, affects ventilation in many ways [17].

The components of disagreement for the statement regarding ARDS pertained to the timing of the disease and the contingency that low tidal volume will potentiate the effect. Various stages of ARDS severity are well-defined, but the timing of developing similar severity levels depends on many factors and differs from patient to patient [18]. The contingency question explicitly referred to the physiology explaining the relationship between tidal volume and dead space. However, the question was likely interpreted as management of ARDS, which is well known to include low tidal volume [19].

This project is unique in establishing expert consensus for respiratory physiology, enabling us to model a digital twin for a critically ill patient in acute hypoxic and hypercapnic respiratory failure. While previous medical applications of AI models have been established for chronic disease management, a model for the ICU has yet to be developed. Existing medical models are limited by a “black box” approach without transparency of how outputs are developed. By relying on causal relationships described by clinicians, our approach is based on interactions of patient physiology rather than large data sets with uncertain associations. This method provides students and clinicians with a model that can clearly display physiology as understood by critical care experts.

Conducting a multinational Delphi research project has its strengths and weaknesses, which are discussed below. A major challenge in conducting a multinational Delphi research project is the language barrier. It may be difficult to find experts who are fluent in a common language, which can make it challenging to communicate effectively throughout the research project. To circumvent this, we maintained a two-way open communication channel with all collaborators to clarify any confusion or misunderstandings related to the expert statements or rules. The survey was administered only in English with the inclusion of terminology which is universally accepted in medicine. As an additional failsafe measure, free text boxes were provided so that survey participants could share their interpretations, opinions, or questions for clarification. Limitations of this study also involve the subjective disposition of collecting data through a survey tool. The first survey round, which included the most questions, required approximately 30 minutes to complete, which limited expert participation. Even though the subsequent rounds had fewer questions with less time required for completion, there still needed to be full participation from the invited experts. Cultural differences can also pose a challenge in a multinational Delphi research project. Different cultures may have different values, beliefs, and attitudes, which can influence the experts’ opinions and may lead to a lack of consensus. Differences in the resource utilization, practice variation can also introduce some bias in the Delphi process. The experts selected for the Delphi research project may not be representative of the entire population and may introduce some sampling bias. We have intentionally tried to minimize this by the inclusion of specialists of both sexes and during the different phases of their careers (early career physicians and more experienced).

However, the international consensus is also a strength of this study. The expert demographics from multiple institutions and various fields of expertise further support this. Conducting a Delphi research project with experts from different countries or regions has provided us with a more diverse range of opinions and perspectives and has led to a more comprehensive understanding of the topic being studied. Including experts from different countries or regions has increased the validity of the results obtained, taking into account the practice variation in different regions of the world and at different levels of experience. This is because the opinions of the experts are based on their experiences and knowledge of the local context, which can provide a more accurate representation of the management of acute respiratory failure in acutely ill patients. The results of a multinational Delphi also increase the generalizability of the findings. Involving experts from different countries and regions has helped us to ensure that the research is culturally sensitive and avoids any bias that may arise from a single country or region perspective and mitigates the risks associated with a smaller sample size concentrated in one geographical area and institution.

These methods and results contribute to the currently available literature by providing expert rule statements for respiratory failure pathophysiology in medical ICU patients with agreement from international content experts. This project further strengthens the concept that the modified Delphi method efficiently establishes agreement on complex physiologic concepts. This allows knowledge of medical experience from the ICU to be applied to strengthen the respiratory component of the ICU digital twin. Similar models have been designed for chronic medical conditions, such as the Archimedes model and diabetes management [2], but a tool for critical care is lacking [6].

The ICU digital twin may benefit undergraduate medical education by providing a medical simulation experience for learners without any patient risk, which can otherwise be challenging in the critical care setting. In the subsequent phases, we will be exploring the usability testing of the digital twin application (including the interventions for management of critically ill patients) [20]. It also facilitates in silico research, where critical care interventions could be researched in virtual patient populations [21]. Research evaluating critical care interventions and patient outcomes presents many challenges, including high-acuity situations that must more easily conform to research protocols. Creating an ICU digital twin and virtual critically ill patient cohort may allow patient outcomes from intensive care admissions to be more thoroughly studied. After validation, predicting intervention outcomes with the ICU digital twin can be used to support clinical decision-making for learners and intensivists.

## Conclusion

In conclusion, conducting a multinational Delphi research project can provide a more diverse range of opinions and perspectives, which can increase the validity and generalizability of the findings. This is much needed in the evolving arena of the development of Digital twin technology. Our study utilizes a modified Delphi methodology to produce expert consensus on acute respiratory failure for an ICU digital twin model. After 3 rounds of Delphi surveys, a multinational cohort of critical care experts reached a consensus on 87% (62 statements) out of the 71 final statements for respiratory failure pathophysiology in the medical ICU. Future work will include translating these agreed-upon expert rules into programmable statements that will be used in clinical simulations to further validate the ICU digital twin model. After acquiring a certain degree of fidelity and multiple cycles of iterative refinement, this model can be used as a clinical decision-support tool at the bedside and for medical education.

### Current knowledge

Artificial intelligence tools have been created and validated for certain chronic medical conditions, such as diabetes. An AI “digital twin” model has been designed and validated for use in the care of septic patients who are critically ill. The ICU digital twin model has not been refined by its specific organ systems, including the respiratory system.

### What this paper contributes to our knowledge

In a modified Delphi design, statements describing the respiratory system pathophysiology of critically ill patients were refined. Consensus was gained by an expert panel for 87% of the Delphi statements. This project demonstrates the use of a modified Delphi as an effective way to refine content for our digital twin model using a causal AI approach.

## Acknowledgments

Data presented at Chest 2022 in Nashville.

**Collaborative authors list:**

This study was performed on behalf of the *Digital Twin Platform for education, research, and healthcare delivery investigator group.*

**Akira Kuriyama, MD, MPH, PhD**, Emergency and Critical Care Center, Kurashiki Central Hospital, Okayama, Japan

**Takuma Minami, MD, PhD,** Department of Primary Care and Emergency Medicine, Kyoto University Graduate School of Medicine, Kyoto Japan

**Tadaaki Takada, MD,** Department of Emergency Medicine and Critical Care, Tokushima Red Cross Hospital, Tokushima, Japan

**Munenori Kusunoki, MD, PhD,** Department of Anesthesiology, Kansai Medical University Hospital, Osaka, Japan

**Tomomi Yoshino, MD,** Emergency and Critical Care Medical Center, Osaka City General Hospital, Osaka, Japan

**Takanao Otake, MD,** Emergency and Critical Care Center, Kurashiki Central Hospital, Okayama, Japan

**Mizuki Sato, MD,** Department of Critical Care Medicine, Shonan Kamakura General Hospital, Kanagawa, Japan

**Yasuhiro Norisue, MD, PhD,** Department of Emergency and Critical Care Medicine/ Department of Pulmonary Medicine, Tokyo Bay Urayasu-Ichikawa Medical Center, Chiba, Japan

**Toshiki Yokoyama, MD, PhD,** Department of Emergency and Intensive Care Medicine, Intensive Care Unit, Tosei General Hospital, Aichi, Japan

**Daisuke Kawakami, MD,** Department of Intensive Care Medicine, Iizuka Hospital, Fukuoka, Japan

**Emiko Nakataki, MD, PhD,** Department of Critical Care Medicine, Tokushima Prefectural Central Hospital, Tokushima, Japan

**Mutsuo Onodera, MD,** Department of Emergency Medicine, Toyota Memorial Hospital, Aichi, Japan

**Rodrigo Cartin-Ceba, MD, MSc,** Pulmonary and Critical Care Medicine, Mayo Clinic, Arizona, USA

**Juan L. Pinedo Portilla,** Medicina Intensiva, Servicio de Cuidados Intermedios Hospital Nacional Almanzor Aguinaga Asenjo, EsSalud, Perú

**Roman Melamed, MD, FCCP,** Abbott Northwestern Hospital, Minneapolis, USA

**Jamie L. Sturgill, PhD,** University of Kentucky, Lexington, USA

**Marija Vukoja, MD, PhD,** Department of Internal Medicine, Faculty of Medicine, University of Novi Sad, The Institute for Pulmonary Diseases of Vojvodina, Sremska Kamenica, Serbia

**Abdullah M. Alhammad, PharmD,** Clinical Pharmacy at the College of Pharmacy, King Saud University (KSU), Riyadh, Kingdom of Saudi Arabia

**Brittany D. Bissell, PharmD, PhD, FCCM, BCCCP,** University of Kentucky, Lexington, USA

**Kshama Daphtary, MD, MBI,** Department of Pediatric Critical Care Medicine, Cleveland Clinic, Cleveland, USA

**Megan A. Rech, PharmD, MS, FCCM,** Loyola University Medical Center Department of Pharmacy and Loyola University Chicago Stritch School of Medicine Department of Emergency Medicine, Maywood, USA

**Michael J. Lanspa, MD,** Intermountain Medical Center Department of Pulmonary and Critical Care, USA
